# Comparison of molecular phenotypes of ductal carcinoma *in situ* and invasive breast cancer

**DOI:** 10.1186/bcr2128

**Published:** 2008-08-05

**Authors:** Rulla M Tamimi, Heather J Baer, Jonathan Marotti, Mark Galan, Laurie Galaburda, Yineng Fu, Anne C Deitz, James L Connolly, Stuart J Schnitt, Graham A Colditz, Laura C Collins

**Affiliations:** 1 Channing Laboratory, Department of Medicine, Brigham and Women's Hospital and Harvard Medical School, 181 Longwood Avenue, Boston, MA, 02115, USA; 2 Department of Epidemiology, Harvard School of Public Health, 677 Huntington Avenue, Boston, MA, 02115, USA; 3 Department of Pathology, Beth Israel Deaconess Medical Center and Harvard Medical School, 330 Brookline Avenue, Boston, MA 02115, USA; 4 Worldwide Epidemiology, GlaxoSmithKline, 1250 S Collegeville Rd, Collegeville, PA, 19426, USA; 5 Department of Surgery, Washington University School of Medicine, 660 S. Euclid Avenue, St. Louis, MO 63110, USA

## Abstract

**Introduction:**

At least four major categories of invasive breast cancer that are associated with different clinical outcomes have been identified by gene expression profiling: luminal A, luminal B, human epidermal growth factor receptor 2 (HER2) and basal-like. However, the prevalence of these phenotypes among cases of ductal carcinoma *in situ* (DCIS) has not been previously evaluated in detail. The purpose of this study was to compare the prevalence of these distinct molecular subtypes among cases of DCIS and invasive breast cancer.

**Methods:**

We constructed tissue microarrays (TMAs) from breast cancers that developed in 2897 women enrolled in the Nurses' Health Study (1976 to 1996). TMA slides were immunostained for oestrogen receptor (ER), progesterone receptor (PR), HER2, cytokeratin 5/6 (CK5/6) and epidermal growth factor receptor (EGFR). Using these immunostain results, cases were grouped into molecularly defined subtypes.

**Results:**

The prevalence of the distinct molecular phenotypes differed significantly between DCIS (n = 272) and invasive breast cancers (n = 2249). The luminal A phenotype was significantly more frequent among invasive cancers (73.4%) than among DCIS lesions (62.5%) (p = 0.0002). In contrast, luminal B and HER2 molecular phenotypes were both more frequent among DCIS (13.2% and 13.6%, respectively) as compared with invasive tumours (5.2% and 5.7%, respectively) (p < 0.0001). The basal-like phenotype was more frequent among the invasive cancers (10.9%) than DCIS (7.7%), although this difference was not statistically significant (p = 0.15). High-grade DCIS and invasive tumours were more likely to be HER2 type and basal-like than low- or intermediate-grade lesions. Among invasive tumours, basal-like and HER2 type tumours were more likely to be more than 2 cm in size, high-grade and have nodal involvement compared with luminal A tumours.

**Conclusion:**

The major molecular phenotypes previously identified among invasive breast cancers were also identified among cases of DCIS. However, the prevalence of the luminal A, luminal B and HER2 phenotypes differed significantly between DCIS and invasive breast cancers.

## Introduction

Recent studies using microarray technology and unsupervised cluster analysis have provided new insights into the classification of invasive breast cancers [1-4]. These studies have resulted in the identification of several breast cancer subgroups that vary in their gene expression signatures and clinical course. The molecularly distinct breast cancer subgroups identified to date include luminal subtypes A and B (both of which are hormone receptor-positive), the HER2 subtype and a group known as basal-like cancers [1-4].

Immunohistochemical staining of paraffin sections using antibody panels has been shown to be a reliable surrogate for molecular classification of invasive breast cancers as categorised by gene expression profiling studies [4-8]. Antibodies against oestrogen receptor (ER), progesterone receptor (PR), human epidermal growth factor receptor 2 (HER2), cytokeratin 5/6 (CK5/6) and epidermal growth factor receptor (EGFR) have been particularly useful for this purpose. In fact, this approach to molecular classification (that is, using immunostaining as a surrogate for expression profiling) is arguably the most practical approach to phenotyping large numbers of archived specimens for which fresh tissue is not available for expression array analysis [9-11]. In addition, application of this method to archival tissues from existing studies provides the opportunity to correlate tissue-marker data with long-term exposures and follow-up data.

While new information regarding the molecular heterogeneity of invasive cancers is rapidly emerging, far less is known about the spectrum of molecular phenotypes among cases of ductal carcinoma *in situ* (DCIS), the immediate precursor to invasive breast cancer. In particular, whether or not the same molecular subtypes identified among invasive breast cancers are also seen in DCIS has not been widely studied [12-15]. The purpose of the current study, therefore, was to determine in a large, well-characterised population of women with invasive breast cancer and DCIS which of the molecular phenotypes found in invasive cancers are also seen in DCIS and the frequency of the various molecular phenotypes in DCIS relative to that in invasive breast cancers.

## Materials and methods

### Study design and population

The Nurses' Health Study was initiated in 1976, with 121,700 US-registered nurses aged between 30 and 55 returning an initial questionnaire. The cohort has been followed by mailed questionnaires biennially to update exposure information and ascertain non-fatal incident diseases. Information on body mass index (BMI), reproductive history, age at menopause and postmenopausal hormone use, as well as diagnosis of cancer and other diseases are updated every two years through questionnaires. The follow-up rate among this cohort was more than 90% through 1996.

### Breast cancer case confirmation

All women reporting incident diagnoses of cancer were asked for permission to review their medical records to confirm the diagnosis and to classify cancers as *in situ* or invasive, by histological type, size, and presence or absence of metastases. To identify cases of cancer in non-respondents who died, death certificates for all deceased participants and medical records for the incident cancers were obtained. Following medical record review, 99% of self-reported breast cancers were confirmed.

### Breast cancer tissue block collection

In 1993, we began collecting archived formalin-fixed paraffin-embedded breast cancer blocks for participants with primary incident breast cancers over 20 years of follow-up (1976 to 1996). Cases who reported a prevalent cancer including breast cancer at baseline were excluded from collection. Of the 5610 patients with breast cancers that were eligible for block collection, we were unable to obtain any pathology material for 1858 cases. The primary reason was because they had been destroyed by the hospital (45%). The majority of hospitals archive tissue blocks for between five and 10 years, therefore we were more successful in obtaining more recent blocks. The year of diagnosis and age at diagnosis were highly correlated (Spearman correlation = 0.49; *p* < 0.0001) and the temporal effect on our collections is evident, not only in the differences in age at diagnosis, but also in the frequency of premenopausal breast cancers when comparing the women from whom we obtained specimens with those for whom we did not. However, these two groups of women were very similar with regards to a number of other breast cancer risk factors and tumour characteristics (Table 1). After taking into account age and year of diagnosis, the participants whose tumours were included in the tissue microarrays (TMAs) were very similar to those for whom we were unable to obtain tissue blocks.

**Table 1:** Comparison of breast cancer risk factors and breast tumour characteristics according to those who were eligible for the study, those for whom we received pathology specimens and those who were included in the tissue microarrays, Nurses' Health Study (1976 to 1996)

	**Eligible for TMA (*n* = 5610)**	**Received pathology specimens (*n* = 3752)**	**Included in TMA (*n* = 2897)**
*Means*			
Age at diagnosis, y	56.4	57.4	57.5
Age at menarche, y	12.4	12.4	12.4
Age at first birth, y	25.4	25.4	25.3
Age at menopause, y	46.9	47.1	47.1
BMI at age 18, kg/m^2^	21.0	21.0	21.0
Parity	3.2	3.2	3.2
Alcohol g/week	6.6	6.1	6.2
			
*Frequencies, n (%)*			
Family history of BC	805 (14.4)	559 (14.9)	409 (14.1)
Nulliparous	430 (7.7)	281 (7.5)	211 (7.3)
Premenopausal	1345 (24.0)	782 (20.8)	600 (20.7)
Prior BBD	2662 (47.5)	1852(49.4)	1360 (47.0)
Current PMH user	1239 (33.8)	884 (34.2)	688 (34.6)
			
Year of diagnosis			
1976 to 1980	517 (9.4)	223 (6.1)	158 (5.6)
1980 to 1985	1066 (19.4)	565 (15.4)	431 (15.2)
1985 to 1990	1678 (30.6)	1152 (31.3)	899 (31.6)
1990 to 1996	2230 (40.6)	1739 (47.3)	1357 (47.7)
			
*Tumour characteristics*^a^			
DCIS	654 (11.7)	443 (11.8)	270 (9.3)
Invasive	4956 (88.3)	3309 (88.2)	2627 (90.7)
			
Tumour size			
Less than 2 cm	3162 (65.6)	2197 (66.7)	1685 (64.9)
More than 2 cm	1656 (34.4)	1098 (33.3)	911 (35.1)
			
Grade			
Well differentiated	319 (15.2)	240 (16.2)	176 (14.7)
Moderately	860 (41.0)	630 (42.4)	515 (43.1)
Poorly	919 (43.8)	615 (41.4)	505 (42.2)
			
Nodal involvement			
No nodes	3931 (70.1)	2684 (71.5)	2006 (69.2)
1 to 3 nodes	904 (16.1)	585 (15.6)	485 (16.7)
4 to 9 nodes	428 (7.6)	268 (7.1)	213 (7.4)
More than 10 nodes	228 (4.1)	140 (3.7)	129 (4.5)
Metastatic	119 (2.1)	75 (2.0)	64 (2.2)
			
Receptor status			
ER+	2738 (74.5)	1953 (75.2)	1572 (75.3)
PR+	2083 (61.2)	1518 (61.9)	1235 (62.3)

^a^Tumour characteristics abstracted from medical pathology reports. BC, breast cancer; BBD, benign breast disease; BMI, body mass index; DCIS, ductal carcinoma *in situ*; ER, oestrogen receptor; PMH, postmenopausal hormone; PR, progesterone receptor; TMA, tissue microarrays.

We obtained pathology samples for 3752 participants. Of these, 390 specimens were only slides stained with haematoxylin and eosin (H&E) and 45 tissue blocks had to be returned to the lending hospital before construction of the TMAs and therefore could not be included. A single pathologist (YF) reviewed H&E sections from eligible cases to confirm the cancer diagnosis, classify the cancer according to histological type and grade (Nottingham), and circle the area from which the cores for the TMAs would be taken. Pathology review identified 420 tumour blocks as being unusable for TMA construction (eg, the block did not contain residual tumour or there was insufficient tumour in the block). TMAs were constructed in the Dana Farber Harvard Cancer Center Tissue Microarray Core Facility, Boston, MA.

Three cores 0.6 mm in diameter were obtained from each breast cancer sample and inserted into the recipient TMA blocks. In total, 23 TMA blocks were constructed from 3093 cancers and positive lymph nodes from 2897 participants. We excluded from the current analysis participants with positive lymph nodes only (*n* = 25), lobular carcinoma *in situ* (*n* = 31), *in situ* carcinomas with both ductal and lobular features (*n* = 13), and additional rare tumour types including malignant phyllodes tumours, neuroendocrine carcinoma and angiosarcoma (*n* = 10). In situ carcinomas with both ductal and lobular features were excluded because their ambiguous histological features precluded their definitive categorisation as either DCIS or lobular carcinoma *in situ*. For participants with both tumour and lymph node tissue, only the tumour tissue was evaluated in the current study. If the invasive case was present with DCIS, the tumour was considered invasive and scored as such.

### Immunohistochemical analysis

We performed immunohistochemical staining for ER, PR, HER2, CK5/6 and EGFR on 5 μm paraffin sections cut from the TMA blocks. Immunostains for each marker were performed in a single staining run on a Dako Autostainer (Dako Corporation). These particular biomarkers were selected for analysis because they are commonly used as a surrogate to classify invasive breast cancers according to their molecular phenotypes [4-8]. Sources and dilutions of the primary antibodies used in this study are listed in Table 2. Immunostaining was conducted according to established protocols. Appropriate positive and negative controls were included in all staining runs.

**Table 2:** Sources and dilutions of primary antibodies used in this study

**Antibody**	**Clone**	**Manufacturer**	**Dilution**
ER	1D5	Dako	1:200
PR	PgR 636	Dako	1:50
HER2	A0485 (rabbit polyclonal)	Dako	1:400
CK5/6	D5/16B4	Dako	1:50
EGFR	2-18C9	Dako	pre-diluted (pharmDX kit)

CK5/6, cytokeratin 5/6; EGFR, epidermal growth factor receptor; ER, oestrogen receptor; PR, progesterone receptor; HER2, human epidermal growth factor receptor 2.

Immunostained TMA slides were evaluated for ER and PR expression, HER2 protein overexpression and expression of CK5/6 and EGFR in each core. Tumour cells that showed nuclear staining for ER or PR were considered ER-positive or PR-positive respectively, whereas all ER-negative or PR-negative cases showed complete absence of tumour cell staining. Of note, low ER-positive or PR-positive (1 to 10% of tumour cell nuclei staining) and ER-positive or PR-positive (>10% of tumour cell nuclei staining) were catagorised as a single "positive" category for the purposes of this analysis. Tumour cells were considered positive for HER2 protein overexpression when more than 10% of the cells showed moderate or strong membrane staining (2+ and 3+). The results of analyses in which HER2 positivity was defined as 3+ were very similar to those presented with a definition of 2+ and 3+. Cases were considered basal CK-positive or EGFR-positive if any cytoplasmic and/or membranous staining was detected in the tumour cells, even if focal. These latter criteria are similar to those previously used for scoring these markers in invasive basal-like cancers [4-6].

### Classification of molecular phenotype

Immunostained TMA sections were reviewed under a microscope and visually scored for each individual tissue core as previously described. We classified a case as positive if there was staining in any of the three cores from that patient and negative if there was no immunostaining present. Cases that were ER-positive and/or PR-positive and HER2-negative were classified as luminal A cancers; cases that were ER-positive and/or PR-positive and HER2-positive were classified as luminal B cancers; cases that were ER-negative, PR-negative and HER2-positive were classified as HER2 type; and cases that were negative for ER, PR and HER2, and positive for CK 5/6 and/or EGFR were categorised as basal-like. Cases that lacked expression of all five markers were considered "unclassified" or "null".

### Statistical analysis

Information on breast cancer risk factors was obtained from questionnaires completed biennially. Covariate data at the time of diagnosis were obtained from the questionnaire before the report of breast cancer diagnosis. Chi-squared tests were used to evaluate the independence of selected variables under the null hypothesis. All statistical tests were two-sided and *p* < 0.05 was considered statistically significant. Informed consent was obtained from each participant. This study was approved by the Committee on the Use of Human Subjects in Research at Brigham and Women's Hospital.

## Results

This analysis population consisted of breast cancers that developed in women participating in the Nurses' Health Study after the baseline questionnaire (in 1976) through to the 1996 follow-up cycle and that could be classified into one of five molecular phenotypes (*n* = 2521; 272 DCIS and 2249 invasive). Based on immunostaining data from the five markers used, overall 1820 tumours were classified as luminal A; 152 were luminal B; 165 were HER2; 266 were basal-like; and 118 tumours were unclassifiable (ER-negative/PR-/HER2-negative/EGFR-negative/CK5/6-negative). An additional 297 cases were excluded because of staining that could not be evaluated or insufficient tumour tissue in the core of the sample.

In general, breast cancer risk factors in women with DCIS and invasive breast cancer were found to be similar (Table 3). Women with DCIS were slightly younger when they first gave birth (24.9 vs. 25.4, *p* = 0.05), were more likely to report a family history of breast cancer (19.0 vs 13.3%; *p* = 0.02) and a previous benign breast disease (57.0 vs 44.8%; *p* = 0.0001) compared with women with invasive breast cancer. As expected, women with DCIS were more likely to report their tumour being detected by screening mammography (81.6%) compared with women with invasive tumours (38.2%).

**Table 3:** Age and age-standardised characteristics of breast cancer cases, Nurses' Health Study (1976 to 1996)

**Risk factors**	**DCIS (*n* = 272)**	**Invasive (*n* = 2249)**
*Means*		
Age at diagnosis, y	57.9	57.3
Age at menopause, y	45.2	45.5
Age at menarche, y	12.3	12.4
Body mass index at age 18, kg/m^2^	20.9	21.0
Parity^a^	3.1	3.2
Age at first birth^a^, y	24.9	25.4
Alcohol, g/week	5.7	6.3
		
*Frequency, %*		
Family history of breast cancer	19.0	13.3
Nulliparous	6.9	6.9
Premenopausal at diagnosis	22.4	20.8
Postmenopausal at diagnosis	76.2	77.2
Prior benign breast disease	57.0	44.8
Current postmenopausal hormone use^b^	37.3	31.9
		
*Molecular Markers, %*		
ER positive	74.0	77.0
PR positive	59.9	64.4
HER2 positive	27.2	10.9

^a^Among parous women only. ^b^Among postmenopausal women only. DCIS, ductal carcinoma *in situ*; ER, oestrogen receptor; HER2, human epidermal growth factor receptor 2; PR, progesterone receptor.

Compared with invasive tumours, DCIS lesions were more likely to be HER2-positive (*p* < 0.0001). The prevalence of the molecular phenotypes differed significantly between DCIS and invasive breast cancers (Table 4). Invasive tumours were significantly more likely than DCIS to be luminal A (*p* = 0.0002). In contrast, luminal B and HER2 molecular phenotypes were more frequent among DCIS than among invasive tumours (*p* < 0.0001). The basal-like phenotype was more frequent among the invasive tumours than among DCIS, although this difference was not statistically significant (*p* = 0.15). However, when the analysis of invasive tumours was restricted to infiltrating ductal carcinomas (*n* = 1550), the frequency of the basal-like phenotype (14.4%) was significantly higher than among DCIS (*p *= 0.005). In an effort to determine if a single marker was responsible for distinguishing invasive tumours from DCIS, we examined ER, PR and HER2 status in multivariate analyses. HER2 was the only marker that significantly distinguished invasive tumours from DCIS (*p* < 0.0001). However, in distinguishing infiltrating ductal tumours from DCIS, ER was also a strong marginally significant predictor (*p* = 0.08).

**Table 4:** Frequency of molecular phenotypes among DCIS and invasive breast cancers, Nurses' Health Study (1976 to 1996)

**Immunophenotype**	**DCIS (*n* = 272)**	**All invasive (*n* = 2249)**	**Infiltrating ductal, NOS only (*n* = 1550)**	***p* value**^a^	***p* value**^b^
	n (%)	n (%)	N (%)		
**Luminal A**	170 (62.5)	1650 (73.4)	1053 (67.9)	0.0002	0.08
**Luminal B**	36 (13.2)	116 (5.2)	90 (5.8)	<0.0001	<0.0001
**HER2+**	37 (13.6)	128 (5.7)	107 (6.9)	<0.0001	<0.0001
**Basal-like**	21 (7.7)	245 (10.9)	223 (14.4)	0.15	0.005
**Unclassified**	8 (2.9)	110 (4.9)	77 (5.0)	0.15	0.14

^a^*p* value from Chi-Squared test comparing DCIS to all invasive tumours.^ b^*p* value from Chi-Squared test comparing DCIS to infiltrating ductal tumours. DCIS, ductal carcinoma *in situ*.

Molecular phenotypes of DCIS and invasive tumours varied according to the grade of the lesions (Table 5). High-nuclear-grade DCIS was significantly more likely than low-grade or intermediate-grade lesions to be HER2 type (*p* < 0.0001) and basal-like (*p* = 0.009). Similarly, high-grade invasive cancers were more likely to be HER2 type (*p* < 0.0001) and basal-like (*p* < 0.0001) than low-grade tumours. Results were similar when the analysis was limited to invasive ductal tumours only.

**Table 5:** Frequency of molecular phenotypes among DCIS and invasive breast cancers according to tumour grade^a^, Nurses' Health Study (1976 to 1996)

**Tumour type**	**Luminal A**	**Luminal B**	**Her2 type**	**Basal**	**Unclassified**
**DCIS**					
DCIS, low nuclear grade	26 (92.9)	1 (3.6)	0	1 (3.6)	0
DCIS, intermediate grade	109 (79.0)	15 (10.9)	6 (4.4)	6 (4.4)	2 (1.5)
DCIS, high nuclear grade	35 (33.0)	20 (18.9)	31 (29.3)	14 (13.2)	6 (5.7)
					
**Invasive tumours**					
Well-differentiated	134 (95.7)	2 (1.4)	0	2 (1.4)	2 (1.4)
Moderately differentiated	344 (79.3)	24 (5.5)	21 (4.8)	31 (7.1)	14 (3.2)
Poorly differentiated	252 (56.8)	20 (4.5)	43 (9.7)	99 (22.3)	30(6.8)

^a^Grade of DCIS and invasive tumours as determined by centralised pathology review. DCIS, ductal carcinoma *in situ*.

Among invasive tumours, molecular phenotypes were differentially associated with prognostic factors (Table 6). Compared with the luminal A subtype, HER2-type tumours were 2.6 (OR = 2.6, 95%CI 1.8 to 3.9) times as likely to be more than 2 cm in size, 3.6 times (OR = 3.6, 95%CI 2.1 to 6.3) as likely to be high grade and twice as likely to have nodal involvement (OR = 2.1, 95%CI 1.5 to 3.1). Similarly, basal-like tumours were more likely to be more than 2 cm in size (OR = 2.0, 95% 1.5 to 2.7), high grade (OR = 5.3, 95%CI 3.5 to 8.1) and have nodal involvement (OR = 1.5, 95%CI 1.3–1.7) when compared with luminal A tumours.

**Table 6:** Odds ratios (95% confidence interval) of prognostic factors among invasive breast cancers according to molecular phenotype, Nurses' Health Study (1976 to 1996)

**Tumour type**	**Luminal A**		**Luminal B**		**Her2 type**		**Basal**		**Unclassified**	
	***n***	**OR**	***n***	**OR (95%CI)**	***n***	**OR (95%CI)**	***n***	**OR (95%CI)**	***n***	**OR (95%CI)**
**Tumour size**^a,b^										
≤ 2.0 cm	1025		54		48		107		57	
>2.0 cm	537	1.0 (Ref)	55	1.9 (1.3 to 2.8)	68	2.6 (1.8 to 3.9)	119	2.0 (1.5 to 2.7)	44	1.4 (0.9 to 2.1)
										
**Tumour grade**^a^										
Low/Intermediate	478		26		21		33		16	
High	252	1.0 (Ref)	20	1.4 (0.8 to 2.6)	43	3.6 (2.1 to 6.3)	99	5.3 (3.5 to 8.1)	30	3.4 (1.8 to 6.4)
										
**Nodal status**^a,b^										
No nodes	1103		72		61		145		70	
Any nodes	547	1.0 (Ref)	44	1.2 (0.8 to 1.7)	67	2.1 (1.5 to 3.1)	100	1.3 (1.0 to 1.7)	40	1.1 (0.7 to 1.6)
										
**Menopausal status**										
Postmenopausal	1289		76		97		187		83	
Premenopausal	334	1.0 (Ref)	35	1.8 (1.2 to 2.7)	27	1.1 (0.7 to 1.7)	51	1.1 (0.8 to 1.5)	25	1.2 (0.7 to 1.8)

^a^Adjusted for age (five-year categories).^ b^Tumour characteristics abstracted from medical pathology reports. CI, confidence interval; OR, odds ratio.

## Discussion

In this large case series, we have shown that by using a panel of five immunostains, DCIS can be classified into five molecularly defined phenotypes that have been described for invasive breast carcinomas. Furthermore, we have shown that the prevalence of the molecularly defined phenotypes differed significantly between DCIS and invasive breast cancers. DCIS were more likely to be of the luminal B and HER2 phenotypes than invasive tumours. HER2 and basal-like phenotypes were common among both high-grade DCIS and high-grade invasive lesions.

These data provide evidence that DCIS and invasive tumours are both molecularly heterogeneous. Our finding of an increased prevalence of luminal B and HER2 molecular subtypes (ie, HER2-positive) in DCIS is consistent with earlier studies demonstrating a higher prevalence of HER2 protein overexpression and gene amplification among DCIS than invasive breast cancers [16-19]. The explanation for the higher prevalence of HER2 overexpression in DCIS compared with invasive carcinoma remains unresolved and several possible explanations have been offered. These include: some HER2-positive DCIS lose HER2 expression when they progress to invasive cancers[19]; invasive carcinomas may arise more frequently from HER2-negative DCIS than from HER2-positive DCIS; there is a bias in mammographically screened populations toward the detection of DCIS lesions that are HER2-positive since these lesions are more frequently associated with comedo necrosis and, in turn, suspicious mammographic microcalcifications that prompt biopsy than non-high-grade DCIS lesions. In support of this explanation, previous studies have shown that screen-detected DCIS is more often due to the presence of linear branching and coarse granular calcifications, as well as DCIS of high nuclear grade and HER2 overexpression than interval DCIS [20,21].

Consistent with previous studies our results indicate that low-grade invasive cancers are more likely to have the luminal A phenotype, whereas high-grade invasive carcinomas are more likely to be HER2-type and basal-like [6]. In addition, both the HER2 and basal-like tumours were significantly more likely to be associated with poorer prognostic factors including larger tumour size, higher grade and nodal involvement. The Carolina Breast Cancer Study (CBCS) also examined molecular phenotypes of invasive tumours classified using the same immunohistochemical markers and categories as used in the current study [22]. Consistent with our study, Carey and colleagues [22] reported that HER2-type tumours (*n* = 33) were more likely to be high grade and have nodal involvement and that basal-like tumours (*n* = 100) were more likely to be high grade relative to luminal A tumours. We observed a strong association between basal-like tumours and nodal involvement, which was not found in the CBCS study. Although information on prognostic and tumour characteristics was not identical between our studies, both support the hypothesis that immunohistochemical classification of invasive tumours is associated with breast cancer prognosis.

We demonstrated a similar relationship between grade and molecular phenotype for DCIS lesions as was observed for invasive tumours. In particular, HER2-type (84%) and basal-like (67%) DCIS lesions were significantly more likely to be high grade than low-grade or intermediate-grade lesions. A similar distribution of molecular phenotypes of DCIS according to grade was reported in the CBCS, with 92% of HER2 DCIS and 84% of basal-like DCIS being high-grade [13]. These data suggest that similar to invasive tumours, molecular classification of DCIS lesions may be important in identifying more aggressive lesions. Additional support for this hypothesis comes from a recent case-control study of women with DCIS, in which 32 women went on to develop subsequent cancer (cases) and 38 did not develop subsequent disease (controls) [23]. This study found that eight of eight DCIS cases expressing high levels of both markers of basal-like subtype, p16 and Ki67, developed a subsequent tumour [23].

The majority of epidemiological studies have reported very similar risk factors for DCIS and invasive breast cancer [16,24]. However, in these epidemiological studies, DCIS and invasive tumours are often considered together as one endpoint or are considered as two distinct groups without further classification according to receptor status. Studies considering invasive breast cancer according to receptor status have identified risk factors specific for certain phenotypes and not others [25,26]. It is likely, however, that the impact of several established risk factors for DCIS and invasive tumours will differ according to molecularly defined phenotypes. Further identification of risk factors for the different molecular phenotype could be of value in risk assessment and prevention.

A limitation of the current study is that we were unable to obtain tissue blocks from all breast cancers arising in this cohort. Our success in doing so was highly correlated with time between diagnosis and initiation of our tissue block collection. Many hospitals destroy paraffin-embedded tissue blocks after five to 10 years. After taking into account the effect of age and year of diagnosis, the women for whom we were able to obtain tumour specimens were very similar to those for whom we were unable to obtain specimens. The primary differences were due to the temporal consequences of the timing of tumour block collection. In addition, the frequency of receptor status positivity among invasive tumours was very similar to other populations suggesting that samples included in this study are representative of the overall US population.

We used immunohistochemical markers as a surrogate to classify breast cancers into the molecular phenotypes defined by expression profiles. While the antibody panel we used in this study has been shown to be a reliable proxy for classification of invasive breast cancers categorised by gene expression [4-8], the correlation is not perfect and there will be some misclassification of these phenotypes. The molecular phenotype categories as defined by the immunohistochemical markers have been shown to be associated with prognostic markers and survival consistent with what has been seen with classification based on RNA expression assays, suggesting that both methods are capturing distinct subgroups [5,22]. Misclassification of phenotypes may underestimate true differences between the subtypes.

Because lesions had to be large enough to yield multiple cores, it is possible that we may have biased our sample towards larger DCIS lesions. In addition, the frequency of DCIS lesions that we were unable to evaluate in the TMA for ER and HER2 staining was 22% and 18%, respectively. These are higher than what was observed for invasive lesions (6% and 7%, respectively), suggesting that the ability to assess DCIS lesions with three replicate cores is less than for invasive tumours. However, the frequency of molecular phenotypes observed in our study is very similar to those reported in the CBCS among DCIS cases (*n *= 245) [13], suggesting that the DCIS cases included in our study are representative of DCIS cases in the US population.

## Conclusion

The introduction of widespread mammographic screening has resulted in a dramatic increase in the diagnosis of DCIS [27]. A better understanding of the progression from DCIS to invasive cancer to avoid over-treatment is an important public health concern. The HER2 and basal-like molecular phenotypes are more common among high-grade DCIS lesions. Because these phenotypes are also associated with poor prognosis among women with invasive tumours, molecular characterisation of in situ tumours may help predict which lesions will progress to invasive tumours. More aggressive treatments could be targeted to the subset of women with DCIS lesions at highest risk of progressing to invasive cancer.

## Abbreviations

BMI = body mass index; CBCS = Carolina Breast Cancer Study; CK5/6 = cytokeratin 5/6; CI = Confidence interval; DCIS = ductal carcinoma *in situ*; EGFR = epidermal growth factor receptor; ER = oestrogen receptor; HER2 = human epidermal growth factor receptor 2; OR = odds ratio; PMH = postmenopausal hormone; PR = progesterone receptor; TMA = tissue microarrays.

## Competing interests

ACD is a full-time employee of GlaxoSmithKline and has received shares of company stock. The Nurses' Health Study investigators (GAC, RMT) received funding from GlaxoSmithKline to conduct biomarker studies on tissue microarrays. However, study investigators acted independently in generating research hypotheses, results and interpreting the data and deciding to submit the paper for publication.

## Authors' contributions

RMT was responsible for data analyses, manuscript preparation and editing. RMT, HJB, JLC, SJS, GAC and LCC made substantial contributions to the study design and to the interpretation of data. LCC, JM, MG, LG and YF were involved in central pathology review, scoring of stains and contributed substantially to manuscript editing. ACD contributed to manuscript editing. All authors read and approved the final manuscript.
